# Dietary Vitamin D and Its Metabolites Non-Genomically Stabilize the Endothelium

**DOI:** 10.1371/journal.pone.0140370

**Published:** 2015-10-15

**Authors:** Christopher C. Gibson, Chadwick T. Davis, Weiquan Zhu, Jay A. Bowman-Kirigin, Ashley E. Walker, Zhengfu Tai, Kirk R. Thomas, Anthony J. Donato, Lisa A. Lesniewski, Dean Y. Li

**Affiliations:** 1 Program in Molecular Medicine, University of Utah, Salt Lake City, Utah, 84112, United States of America; 2 Department of Bioengineering, University of Utah, Salt Lake City, Utah, 84112, United States of America; 3 Recursion Pharmaceuticals, LLC, Salt Lake City, Utah, 84108, United States of America; 4 Department of Human Genetics, University of Utah, Salt Lake City, Utah, 84112, United States of America; 5 Division of Geriatrics, Department of Medicine, University of Utah, Salt Lake City, Utah, 84112, United States of America; 6 The Key Laboratory for Human Disease Gene Study of Sichuan Province, Institute of Laboratory Medicine, Sichuan Academy of Medical Sciences & Sichuan Provincial People’s Hospital, Chengdu, Sichuan, 610072, China; 7 Division of Cardiology, Department of Medicine, University of Utah, Salt Lake City, Utah, 84112, United States of America; 8 Department of Oncological Sciences, University of Utah, Salt Lake City, Utah, 84112, United States of America; 9 Cardiology Section, VA Salt Lake City Health Care System, Salt Lake City, Utah, 84112, United States of America; University of Alabama at Birmingham, UNITED STATES

## Abstract

Vitamin D is a known modulator of inflammation. Native dietary vitamin D_3_ is thought to be bio-inactive, and beneficial vitamin D_3_ effects are thought to be largely mediated by the metabolite 1,25(OH)_2_D_3_. Reduced serum levels of the most commonly measured precursor metabolite, 25(OH)D_3_, is linked to an increased risk of multiple inflammatory diseases, including: cardiovascular disease, arthritis, multiple sclerosis, and sepsis. Common to all of these diseases is the disruption of endothelial stability and an enhancement of vascular leak. We previously performed an unbiased chemical suppressor screen on a genetic model of vascular instability, and identified cholecalciferol (D_3_, dietary Vitamin D_3_) as a factor that had profound and immediate stabilizing and therapeutic effects in that model. In this manuscript we show that the presumed inactive sterol, D_3_, is actually a potent and general mediator of endothelial stability at physiologically relevant concentrations. We further demonstrate that this phenomenon is apparent in vitamin D_3_ metabolites 25(OH)D_3_ and 1,25(OH)_2_D_3_, and that the effects are independent of the canonical transcription-mediated vitamin D pathway. Our data suggests the presence of an alternative signaling modality by which D_3_ acts directly on endothelial cells to prevent vascular leak. The finding that D_3_ and its metabolites modulate endothelial stability may help explain the clinical correlations between low serum vitamin D levels and the many human diseases with well-described vascular dysfunction phenotypes.

## Introduction

There exists an inverse correlation between measured vitamin D levels and the pathology or incidence of the leading causes of death: cardiovascular disease, stroke, chronic obstructive pulmonary disease (COPD), infection, and cancer [1–9]. Vitamin D deficiency is also linked to multiple autoimmune diseases including multiple sclerosis, arthritis, lupus, and type-1 diabetes [10–15]. Although the classical role of Vitamin D is to maintain calcium homeostasis and promote bone health, more recent studies have shown that Vitamin D, acting through immune cells, induces an anti-inflammatory response, providing a plausible link to many of the aforementioned diseases [16].

A hallmark of inflammation is the activation and destabilization of the endothelial cells lining the vasculature, leading to dysfunctional nutrient exchange, inflammatory cell migration, and dysregulated activation of the clotting cascade [17, 18]. Endothelial destabilization and activation occurs as a result of injury, altered hemodynamics, response to cytokines or other inflammatory cues, as well as genetic diseases [19, 20]. Therapies designed to stabilize the vascular endothelium have been shown to limit the pathology from a diverse array of inflammatory diseases including infection, arthritis, cancer, retinopathy and more [21–25]. Our studies to identify chemical suppressors of the phenotype of one such genetic disease, cerebral cavernous malformation (CCM), identified vitamin D_3_ (D_3_, cholecalciferol, calciol, ‘dietary vitamin D_3_’) as a factor that rescued the destabilized vasculature of CCM *in vitro* and *in vivo* [26].

D_3_ is ingested in foods such as fish, and is also synthesized endogenously by the action of ultraviolet light on 7-dehydrocholesterol (7-DHC) in the skin^14^. D_3_ is hydroxylated to 25-hydroxy vitamin D_3_ (25(OH)D_3_, calcidiol, ‘prohormonal vitamin D_3_’) in the liver by CYP2R1 [27]. 25(OH)D_3_ is further hydroxylated to 1α,25-dihydroxy vitamin D_3_ (1,25(OH)_2_D_3_, calcitriol, ‘hormonal vitamin D_3_’) by CYP27B1 in the kidney [28]. Both D_3_ and 25(OH)D_3_ are widely considered to be biologically inactive, serving only as precursor metabolites of the active form 1,25(OH)_2_D_3_. 1,25(OH)_2_D_3_, a hormone vital to calcium homeostasis and the immune response, acts through the well-characterized nuclear hormone receptor Vitamin D Receptor (VDR) [29]. Having observed endothelial stabilizing activity of D_3_ in CCM2 deficient cells, we wanted to determine if D_3_ and its metabolites are able to serve as general modulators of endothelial stability.

Herein, we show that the previously assumed inactive sterol, vitamin D_3_, is a potent and general mediator of endothelial stability at physiologically relevant levels. We observe this stabilizing effect with D_3_ and the metabolites 25(OH)D_3_ and 1,25(OH)_2_D_3_), but not the vitamin D precursor 7-dehydroxy cholesterol (7-DHC). We also show that the stabilizing effect is broad as it inhibits permeability induced by diverse pro-inflammatory cues. Finally, we determine that this effect is non-genomic, and that it occurs in conjunction with the deactivation of ARF6, RhoA, and the stabilization of VE-Cadherin at the plasma membrane.

The prevalent hypothesis to explain the inverse correlation between vitamin D status and diverse diseases is that 1,25(OH)_2_D_3_ acts directly on immune cells through the vitamin D receptor (VDR) to modulate the immune response [30]. Our data identify an additional role for vitamin D in which the vitamin D sterols act directly on the endothelium to stabilize barrier structure and function, thereby reducing vascular leak into the surrounding tissues. This new observation may explain, in part, the broad associations between vitamin D and many diseases.

## Materials and Methods

### Ethics Statement

All mouse experiments were approved by the University of Utah Institutional Animal Care and Use Committee and the George E. Wahlen Department of Veterans Affairs Medical Center Institutional Animal Care and Use Committee.

### Cells and Culture Conditions

Primary dermal human microvascular endothelial cells (HMVEC-D) were purchased from Lonza at passage 0 (p0), grown in EGM2-MV (Lonza) and experiments were performed from p4-p7 in EBM2 supplemented with 0.1% BSA.

### Trans-endothelial resistance (TEER)

The ECIS system was used with 8-well plates (8W10E+, Applied Biophysics), with cells seeded at 2.5x10^4^ cells/well. Transcription and translation inhibition experiments were performed using a Roche SP Xcelligence transendothelial system and 96-well assay plates with cells seeded at 1x10^4^ cell/well. Treatment in all assays, unless otherwise denoted were 10μm D3 (Tocris), 7-DHC (Sigma) or vehicle (0.5% DMSO) and simultaneous cytokine addition of either TNF-α (2ng/mL) or IL-1β (10ng/mL).

### Transwell permeability

HMVEC-D cells were seeded on 1.0μm BD FalconTM Cell-Culture Inserts coated with human fibronectin. Cells were grown to confluence and pre-treated with 10μm D3 (Tocris), 7-DHC (Sigma) or vehicle (0.5% DMSO) on both apical and basolateral sides of the monolayer for 30 minutes. Cytokines, TNF-α (2ng/mL) or IL-1β (10ng/mL), were then added to both the apical and basolateral sides of the monolayer. Four hours following cytokine addition, a FITC-dextran (40kDa) reporter was added to each upper chamber, reaching a final concentration of 1mg/mL. The solutions were removed from each bottom chamber 60 minutes following FITC-dextran addition. The concentrations of FITC-dextran in the lower chamber solutions were measured using fluorimetry (excitation wavelength = 485nm; emission measured at 530nm) and concentrations were calculated via standard curve. Data are presented as a mean ± SEM of three independent experiments, with four replicates per condition in each experiment.

### Ex-vivo cerebral artery permeability

Mice were euthanized by exsanguination via cardiac puncture while under isoflurane anesthesia. The brain was excised and placed in a cold physiological salt (pH 7.4 @ 4 C) and ~100 μm diameter middle cerebral arteries were dissected and placed in a pressure myograph (DMT Inc) containing physiological salt solution (PSS, 145.0 mM NaCl, 4.7 mM KCl, 2.0 mM CaCl2, 1.17 mM MgSO4, 1.2 mM NaH2PO4, 5.0 mM glucose, 2.0 mM pyruvate, 0.02 mM EDTA, 3.0 mM MOPS buffer and 1 g/100 ml BSA, pH 7.4. Arteries were cannulated onto glass micropipettes, secured with nylon (11–0) suture and perfused with PSS. Once cannulated, the middle cerebral arteries were checked for leaks, warmed to 37C, pressurized to 70 mmHg and allowed to equilibrate for ~1 hour. 70 kD FITC labeled dextran (.14 mg/ml) in PSS was infused intraluminally. Vessels that leaked upon initial inspection were excluded. The artery was protected from the light and 100 μl samples were collected from the bath surrounding the cannulated artery at 0, 1, 2, 2.5 and 3 hours after VEGF treatment (2.5 X 10^-5^mg/ml). FITC fluorescence measures were made in collected PSS via a Synergy 4 plate reader (BioTek) and are expressed in relative fluorescent units (RFU) and normalized to the RFU for the physiological saline.

### Western blot and Phosphorylation assays

Human dermal microvascular endothelial cells (HMVEC-D) were grown to confluence and starved in Endothelial Cell Basal medium-2 (EBM-2) + 0.1% BSA for 4 hours. Then the cells were stimulated with 10ng/ml IL-1β with 10μm D3 (Tocris), 7-DHC (Sigma) or vehicle (0.5% DMSO) for 10 min, or 2ng/ml TNFα with 10μm D3 (Tocris), 7-DHC (Sigma) or vehicle (0.5% DMSO) for 15 min. After treatment, the cells were washed with ice-cold PBS and lysed in 50mM Tris pH 7.4, 150mM NaCl, 10mM MgCl2, 10% Glycerol, 1% NP-40, with protease and phosphatase inhibitors (Roche). Lysates were combined with 2X sample buffer, separated by SDS-PAGE and probed with antibodies to phospho-VE-cadehrin Y731 (Invitrogen), phospho-Src Y418 (Cell signaling), VE- cadherin (Cell signaling), Src (Cell signaling), FoxO1 (Cell signaling) and CYP24 (Thermo) at 1:1000. For RhoA, ARF6, Rac1 /cdc42 and R-Ras activation assays, crude total cell lysate were generated and GTP-RhoA, ARF6, Rac1/ cdc42 and R-Ras were precipitated with Rhotekin-RBD (Millipore), GGA3-PBD (Cell Biolabs), PAK-1-PBD (Millipore) and Raf-1 RBD respectively. Following three washes with lysis buffer, bound proteins were eluted with 2X sample buffer. RhoA, ARF6, Rac1 /cdc42 and R-Ras was detected by western blotting with antibodies (RhoA, Rac1 and R-Ras antibody are from Cell Signaling, ARF6 and cdc42 antibody are from Millipore). Each blot is representative of at least three independent experiments, for which densitometry is shown.

### VE-Cadherin area quantification

10,000 HMVEC per well were seeded on BD-Falcon clear-bottom 96-well tissue culture plates (catalog number 353219) and allowed to grow for 48–72 hours. Cells were treated with denoted molecules for 24 hrs and then fixed with 4% paraformaldehyde in PBS for 10 min. Plates were then blocked with Odyssey™ Blocking Buffer (Licor Biosciences) for 30 min at room temperature, and then a mouse monoclonal VE-Cadherin antibody (Cell Signaling clone D87F2, Cat #2500) over night at 4°C. Cells were rinsed 3X with PBS and incubated with Alexa Fluor^®^ 488 donkey anti-mouse secondary and Hoechst 33258 dye for 4 hr at room temperature. Cells were rinsed and the Molecular Devices ImageXpress Micro XLS collected images at the center of each well with a 20X objective. VE-cadherin area of each image was quantified with ImageJ by batch thresholding all images from an individual experiment and quantifying the number of pixels that were VE-Cadherin positive within each image. Data are compiled from three independent experiments with at least three replicate wells per condition in each experiment.

## Results

### D_3_ and its metabolites stabilize vascular endothelial cells

**Fig 1:**
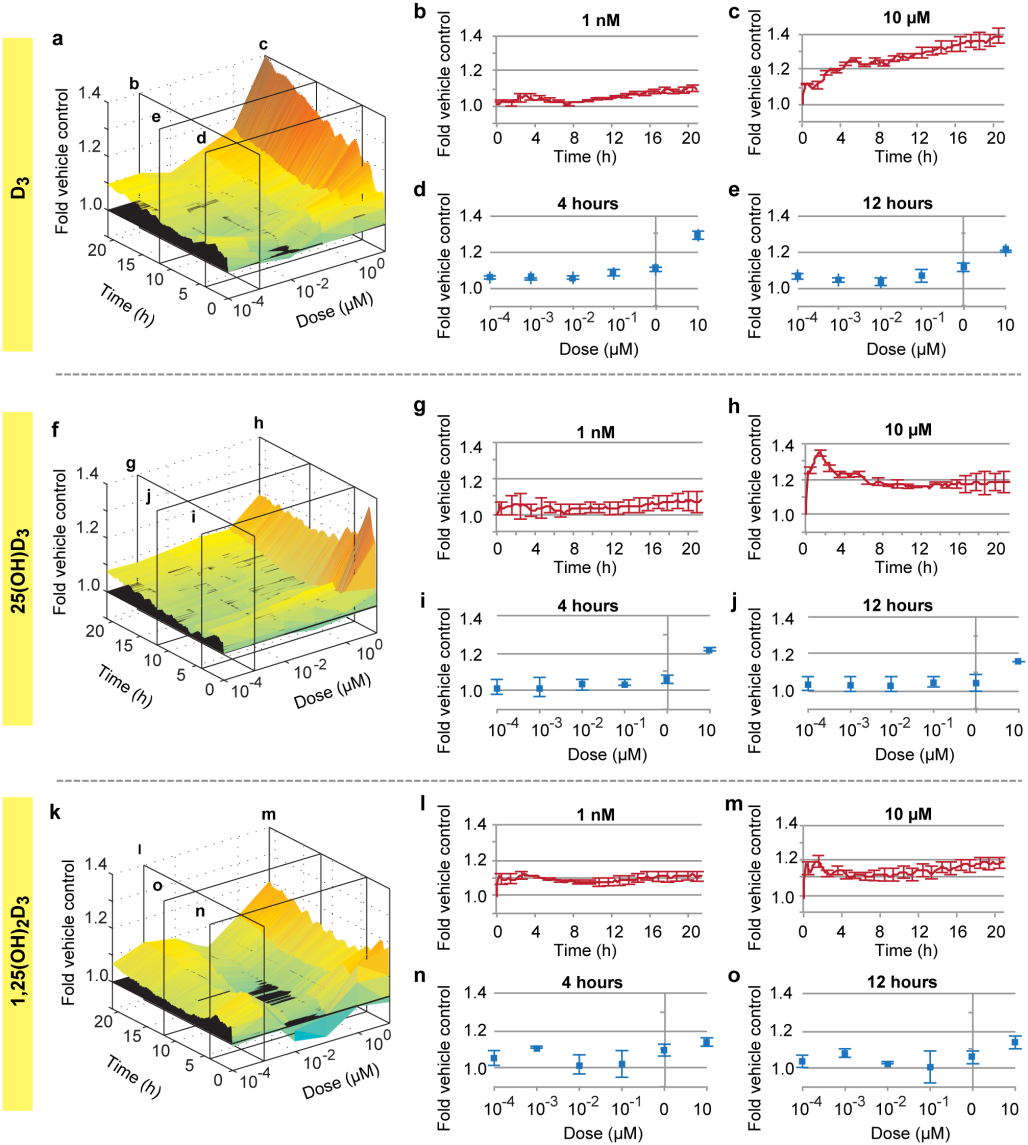
Vitamin D stabilizes the endothelium. Dose/time resistance (endothelial stability) surfaces generated with ECIS are shown from 100 pM to 10 μM and from zero to 21 hours for: (**A**) D_3_; (**F**) 25(OH)D_3_; (**K**) 1,25(OH)_2_D_3_. Detailed time-responses are shown at 1 nM and 10 μM respectively for: (**B** and **C)** D_3_; (**G** and **H**) 25(OH)D_3_; and (**L** and **M**) 1,25(OH)_2_D_3_. Detailed dose-response are shown at 4 hours and 12 hours respectively for (**D** and **E)** D_3_, (**I** and **J**) 25(OH)D_3_, and (**N** and **O**) 1,25(OH)_2_D_3_.

Although vitamin D levels are known to correlate with a variety of indicators of health, this relationship is thought to be mediated through the direct action of 1,25(OH)_2_D_3_ in inflammatory cells [10, 30]. However, we previously found that dietary vitamin D_3_ directly alleviated stability defects in a cellular model of CCM disease [26]. However, because D_3_ is presumed to be biologically inactive, we sought to characterize the stabilizing effects of all three basic forms of vitamin D (D_3_, 25(OH)D_3_, and 1,25(OH)_2_D_3_) in primary human microvascular endothelial cells. Electric cell-substrate impedance sensing (ECIS), an indirect measure of monolayer integrity, was used initially to characterize the stability of endothelial monolayers in the presence of various Vitamin D_3_ related compounds. When administered to the apical side of endothelial monolayers, all three forms of vitamin D_3_ increased baseline endothelial stability within minutes (Fig 1A–1O). We also observed statistically significant activity for all three metabolites at doses as low as 100pM, commensurate with documented circulating levels of 25(OH)D_3_ and D_3_ [31]. Surprisingly, the purportedly inactive dietary form, D_3_, was the most potent of the three sterols across the range of doses from 100 pM to 10 μM.

**Fig 2:**
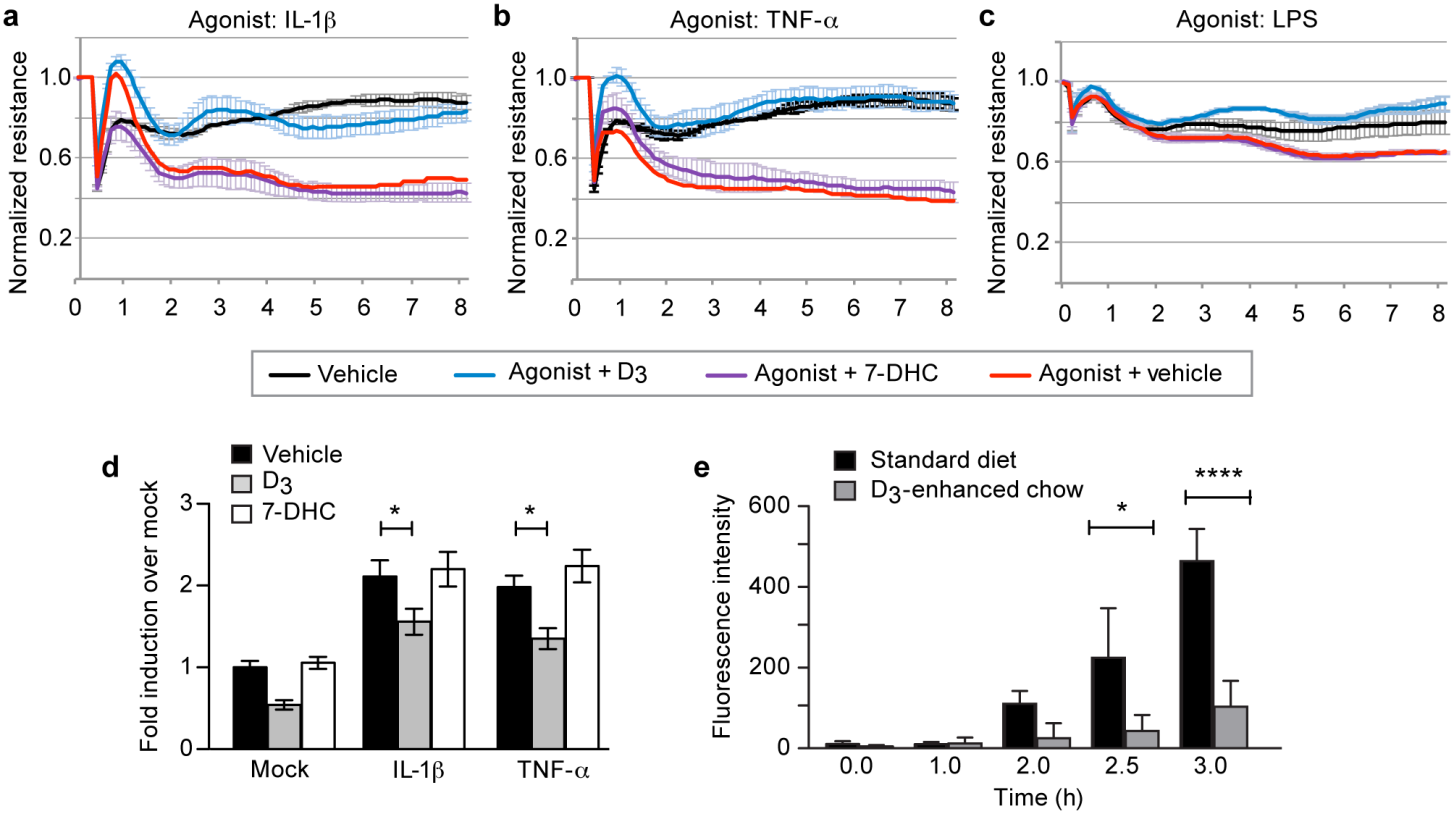
D_3_ abrogates inflammatory leak in culture and *ex* vivo. Monolayers of HMVEC were stimulated with D_3_ (10 μM), 7-DHC(10 μM), or 0.5% DMSO (vehicle control) in the presence of inflammatory cytokines: IL-1β (10 ng/mL), TNF-α (2 ng/mL), and LPS (100 ng/mL) in an (**A-C**) ECIS or (**D**) transwell leak assay. (**E**) VEGF-induced leak of a fluorescent reporter in arterioles isolated from wild-type mice fed either standard chow or a D_3_-enhanced chow. All panels depict mean ± SEM. * denotes P<0.05, ** denotes P<0.01, and **** denotes P<0.0001.

Given the basal modulatory effects on the endothelium, we next asked if D_3_ could exhibit protective effects in the face of pro-inflammatory cues. We challenged endothelial monolayers *in vitro* in the presence of D_3_ with the inflammatory signals, interleukin-1β (IL-1β), tumor necrosis factor-α (TNF-α), or bacterial lipopolysaccharides (LPS) (Fig 2A–2C). ECIS demonstrated that D_3_, but not its precursor 7-dehydrocholesterol (7-DHC), inhibited the destabilizing effects of all of these diverse signals. Because the ECIS readout may also reflect changes in cell shape and cell-substrate adhesion, we confirmed this observation in a transwell assay, a direct measure of monolayer leak, and observed less flux of a 40kD FITC dextran reporter from the apical to basolateral side across endothelial monolayers (Fig 2D). Finally, to determine whether the broad stabilizing effects of D_3_ on the endothelium translated to an animal model, we examined cerebrovascular leak in wild-type mice fed either standard chow (1.5 IU/g D_3_) or an identical chow enhanced with D_3_ (25 IU/g). We found that cerebral arterioles isolated from mice fed a diet high in D_3_ had significantly less VEGF-induced leak of a fluorescent reporter from the lumen through the vessel wall compared to mice fed a standard diet (Fig 2E). These data suggest that the vitamin D sterols act directly on the endothelium to suppress the destabilizing actions of diverse environmental stimuli.

### D3 blocks RhoA and ARF6 activation in endothelial cells

**Fig 3:**
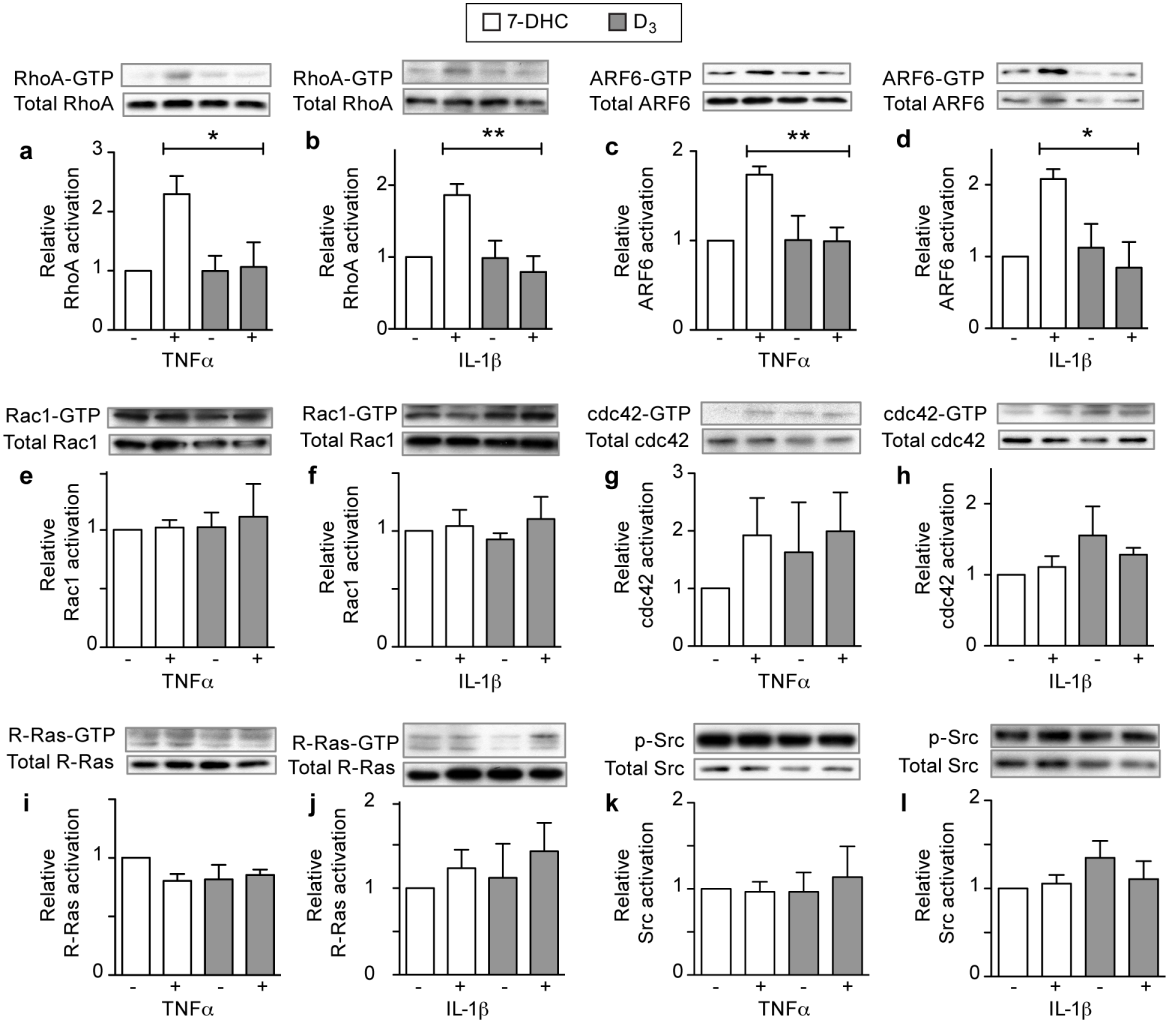
D_3_ blocks RHOA and ARF6 activation in destabilized endothelial cells. Endothelial cells were exposed to 10 μM D_3_ or 7-DHC in combination with 2ng/mL TNF-α or IL-1β. Lysates were analyzed for RHOA-GTP and ARF6-GTP levels using appropriate precipitation assays. All graphs depict mean ± SEM. * denotes P<0.05, ** denotes P<0.01, and *** denotes P<0.001.

To determine the mechanism of D_3_’s activity in endothelial cells, we assessed the activation of markers within the key signaling pathways involved in endothelial stability: transforming protein RhoA (RHOA), Ras-related C3 botulinum toxin substrate 1 (RAC1), cell division control protein 42 homolog (CDC42), ADP-ribosylation factor 6 (ARF6), proto-oncogene tyrosine-protein kinase Src (SRC), or Ras-related protein R-Ras (RRAS) [21, 22, 32–35]. Treatment of HMVEC with D3 suppresses both TNF-α or IL-1β activation of RHOA and ARF6 (Fig 3A–3D and S1 Fig). D_3_ did not modulate the baseline activation of markers left unaltered by TNF-α or IL-1β (Fig 3E–3l and S1 Fig). Taken together, these data suggest that D_3_ can rapidly affect a subset of key intracellular signaling pathways that play a role in endothelial activation in the context of cytokine-induced destabilization.

### D3 stabilizes junctional VE-cadherin

**Fig 4:**
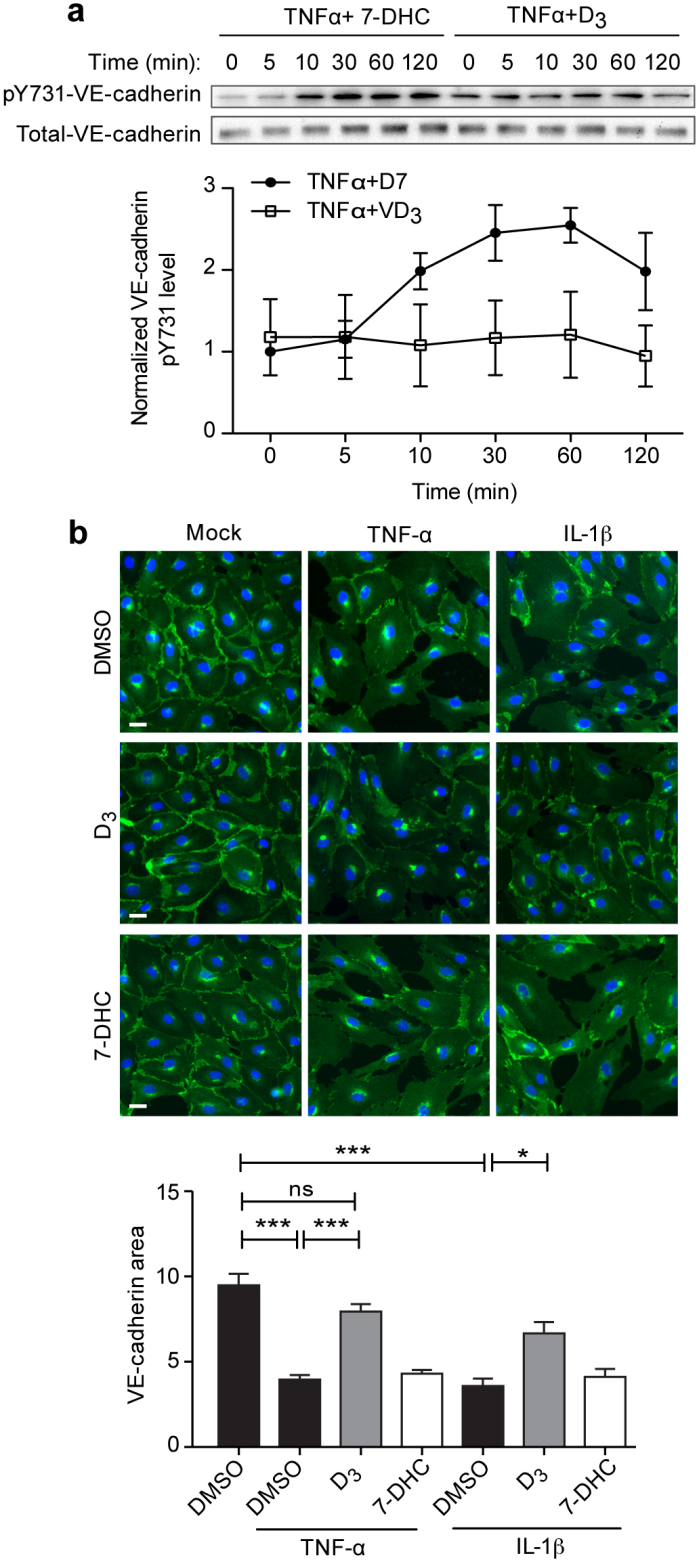
D_3_ promotes VE-cadherin cell-cell junction stability. (**A**) Endothelial cells were treated with TNF-α and either 7-DHC or D_3_ for the denoted times and lysates were immunoblotted for p731 VE-cadherin or total VE-Cadherin. (**B**) Endothelial cell monolayers were exposed to the denoted pro-inflammatory cues in the presence of vehicle control, D_3_ or 7DHC. Cells were fixed and VE-Cadherin was visualized through immunofluorescent labeling with automated image acquisition and analysis. All graphs depict mean ± SEM. * denotes P<0.05, ** denotes P<0.01, and *** denotes P<0.001.

Activated ARF6 (ARF6-GTP) has been shown to play a key role in the destabilization of vascular endothelial cadherin (VE-Cadherin) cell-cell junction proteins, resulting in endothelial monolayer disruption [21, 22]. This activity of ARF6 is mediated through the phosphorylation of tyrosine 731 (pY731) on VE-Cadherin, which destabilizes cadherin-cadherin interaction and leads to VE-Cadherin internalization [36, 37]. Not only did D_3_ inhibit the TNF-α-induced phosphorylation of VE-cadherin, (Fig 4A and S2 Fig), it strengthened the integrity of cell-cell junctions, measured immunocytochemically by VE-Cadherin quantification, between cells exposed to IL-1β and TNF-α (Fig 4B). Collectively, these data suggest that D_3_ may inhibit the destabilizing effects of cytokines on cell-cell junctions through a common node.

### D_3_ acts on endothelial cells through a non-genomic mechanism

**Fig 5:**
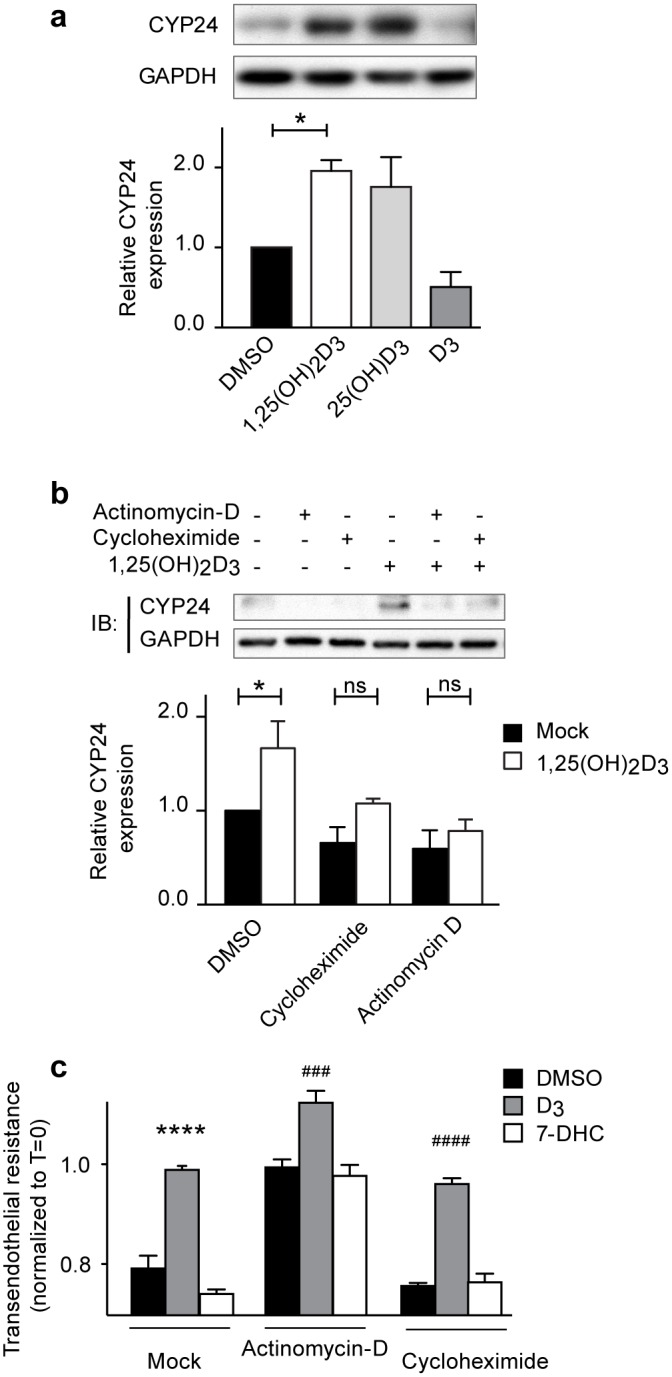
D_3_ stabilizes endothelial cells through a non-genomic mechanism. **(A)** Endothelial cells were exposed to D_3_ or its metabolites for 24 hours and lysates were probed for VDR transcription targets FOX01 and CYP24. Endothelial cells were exposed to D_3_ or its metabolites in the presence of inhibitors of transcription (actinomycin D) and translation (cycloheximide) **(B, C)** and were assessed for transendothelial resistance or VDR target gene expression. All graphs depict mean ± SEM. * denotes P<0.05, and **** denotes P<0.0001. ### denotes P<0.001, and #### denotes P<0.0001 versus the respective control.

1,25(OH)_2_D_3_ is the high-affinity ligand for the nuclear hormone receptor VDR [29, 38–41]. VDR is a transcription factor, though it can also act rapidly via non-transcriptional mechanisms to modulate calcium influx and activate protein kinase c [29, 38, 39, 42]. D_3_ is not considered to be a ligand of VDR, except at non-physiologic doses, and fails to activate known VDR-dependent pathways [29, 39, 41]. 25(OH)D_3_ is also generally considered not to be a high-affinity ligand of VDR, however, in some reports it has been observed to be an agonist of the receptor [29, 38, 39, 41, 43]. Given the considerable evidence suggesting the biologic inactivity of D_3_ and 25(OH)D_3_ in the endothelium, we wondered whether the stabilizing effects of these purportedly inactive compounds could be explained by endothelial-mediated conversion to 1,25(OH)_2_D_3_. To determine whether D_3_ was being converted into 1,25(OH)_2_D_3_, we measured the ability of D_3_ and 25(OH)D_3_ to enhance expression of CYP24A1, a gene that has been reported to be highly expressed as result of 1,25(OH)_2_D_3_ induced VDR activation [44, 45]. Despite the rapid (within minutes) effects of D_3_ in our previous assays, treatment of HMVEC with D_3_ for 24 hours had no measurable effect on expression of VDR target CYP24A1, suggesting D_3_ was not converted to 1,25(OH)_2_D_3_ (Fig 5A). Confirming the validity of the assay, treatment with 1,25(OH)_2_D_3_ led to a significant increase in CYP24A1 expression after a 24 hour treatment. 25(OH)D_3_ induced an increase in CYP24A1, consistent with reports of endothelial conversion of 25(OH)D_3_ to 1,25(OH)_2_D_3_, but not of conversion of D_3_ to 1,25(OH)_2_D_3_ [46]. The observation that all three of the vitamin D sterols exhibit a stabilizing effect, but only two induce the expression of VDR target genes, suggests that the sterols stabilize the endothelium in a manner indpendent from canonical, genomic vitamin D receptor signaling.

1,25(OH)_2_D_3_ is reported to have both genomic and non-genomic activities in multiple tissue and cell types [38, 39]. To test if the effects of D_3_ were genomic and dependent upon *de novo* gene expression, we examined the effect of D_3_ on trans-endothelial resistance in the presence of transcription and translation inhibitors (actinomycin-D and cycloheximide, respectively), and found that while these inhibitors blocked the production of the VDR-target CYP24, they had no effect on the stabilizing effect of D_3_ on the endothelium (Fig 5B and 5C). These data suggest D_3_ has an immediate and direct stabilizing effect on the endothelium, which is not the result of conversion to 1,25(OH)_2_D_3_, transcription, or translation.

## Discussion

Our data show that vitamin D_3_ and its metabolites, 25(OH)D_3_, and 1,25(OH)_2_D_3_, can modulate endothelial stability even in the face of diverse inflammatory cues such as cytokines and lipopolysaccharides. This stabilizing effect, combined with our prior observation that D_3_ preserves vascular integrity in cells deficient in CCM2, suggests there may exist a novel function for vitamin D_3_ in homeostasis by directly maintaining or enhancing the barrier function of the endothelium. Importantly, we also show that the barrier-enhancing function of vitamin D_3_ is not limited to what is commonly referred to as the “active metabolite” 1,25(OH)_2_D_3_, but this activity is present in the “inactive” dietary and mono-hydroxylated forms of the vitamin as well.

Vitamin D has received much attention over the last decade due to the inverse correlation between serum levels of 25(OH)D_3_ and the leading causes of death: cardiovascular disease, stroke, COPD, infection, cancer, as well a number of autoimmune diseases [29, 47]. Vascular instability is a hallmark of such inflammatory diseases, and we and others have previously demonstrated that prevention of vascular destabilization reduces pathology in inflammatory diseases arthritis and septic shock [21–23, 48–50]. In many of the reports characterizing a relationship between 25(OH)D_3_ and disease, D_3_ concentration is ignored and investigators have presumed that 25(OH)D_3_ is not itself active in these disease states [29, 47]. Instead, the assumption has typically been that serum 1,25(OH)_2_D_3_ levels mirror serum 25(OH)D_3_, or that 25(OH)D_3_ is locally converted to 1,25(OH)_2_D_3_ in tissues implicated in disease progression [28, 29, 47]. In many cases, however, these assumptions go untested, and such a mechanism may not be entirely responsible for the beneficial effects of vitamin D.

**Fig 6:**
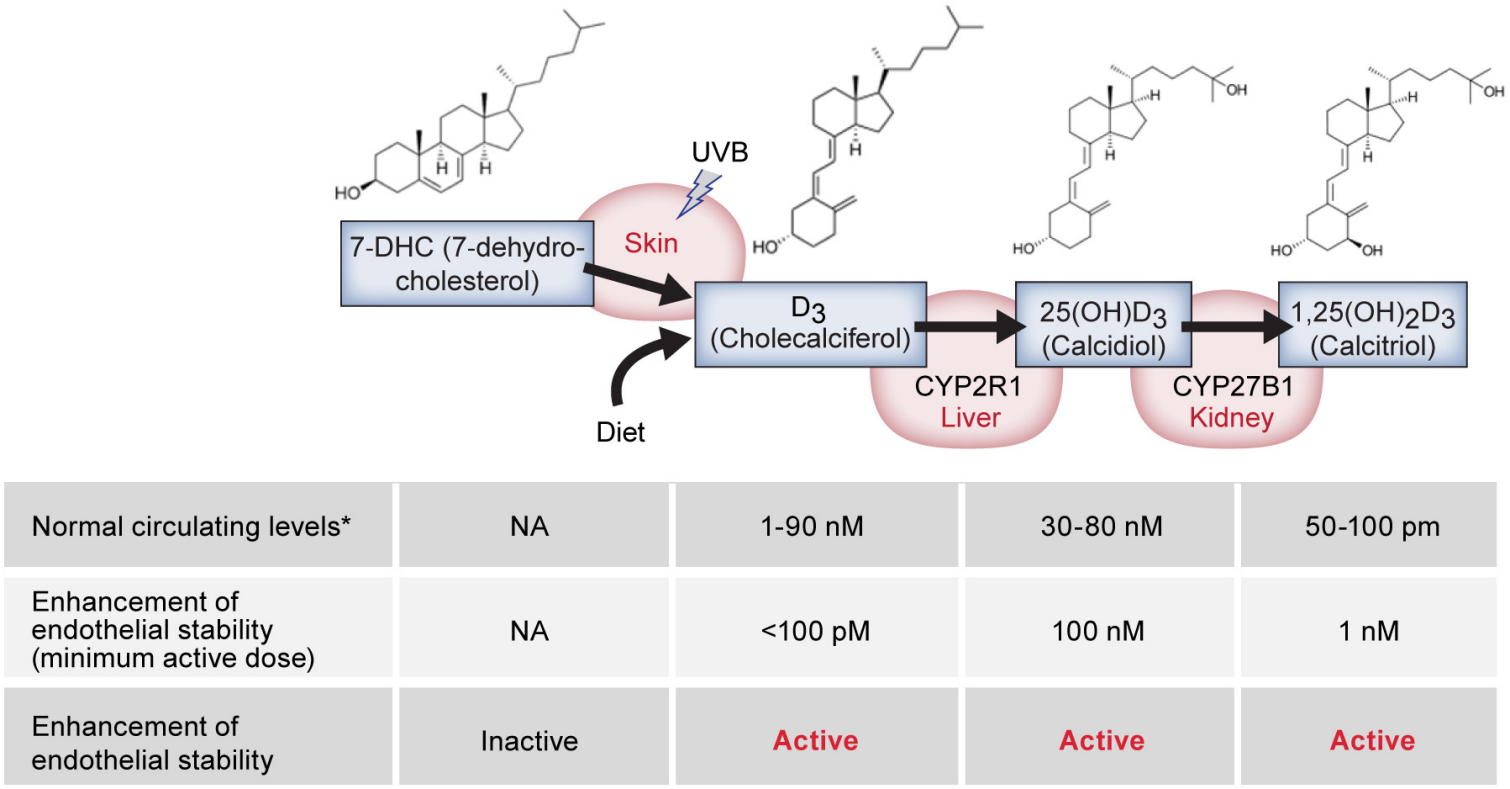
Vitamin D sterol activity. Graphical models of the different vitamin D3 sterols, their metabolism, and a summary of their normal circulating levels, the minimum active dose for stabilizing the endothelium and doses in which the sterols have been reported to interact with vitamin D receptor [29, 51, 52]. *Normal circulating levels vary upon many conditions including diet and UV exposure.

We have thus far described a novel function for vitamin D and its metabolites by which they are similarly bioactive and promote immediate, direct, and stabilizing effects on the endothelium that counter the disruptive effects of inflammatory cues (Fig 6). All three vitamin D metabolites tested in our assays have similar potency in mediating endothelial stability. In humans, circulating serum levels of D_3_ and 25(OH)D_3_ are present in the nanomolar range, approximately 1,000 fold higher than 1,25(OH)_2_D_3_[28]. These data raise the possibility that the traditionally ‘inactive’ metabolites D_3_ and 25(OH)D_3_ might play more prominent roles in controlling endothelial stability and disease compared to 1,25(OH)_2_D_3_, which is not reported to circulate at levels necessary for the observed stabilizing phenomena. Additionally, because the *‘inactive’* sterols can promote stabilizing activity at doses lower than necessary for an interaction with VDR, the stabilizing phenomena may occur in a VDR-independent manner. While this study does not call into question a role for 1,25(OH)_2_D_3_ or VDR in immunomodulatory mechanisms, our results illuminate an alternative pathway of vitamin D activity, and raise the interesting possibility that inverse correlations between serum 25(OH)D_3_ levels and certain diseases could be due to the direct effects of D_3_ and 25(OH)D_3_ on endothelial stability.

## Supporting Information

S1 Fig D_3_ blocks RHOA and ARF6 activation in destabilized endothelial cells (western blot replicates). Endothelial cells were exposed to 10 μM D_3_ or 7-DHC in combination with 2ng/mL TNF-α or IL-1β. Lysates were analyzed for RHOA-GTP and ARF6-GTP levels using appropriate precipitation assays.
(TIF)

S2 Fig D_3_ promotes VE-cadherin cell-cell junction stability (western blot replicates). Endothelial cells were treated with TNF-α and either 7-DHC or D_3_ for the denoted times and lysates were immunoblotted for p731 VE-cadherin or total VE-Cadherin.
(TIF)
