# Supplementary figures and images for: Dietary Vitamin D and Its Metabolites Non-Genomically Stabilize the Endothelium
Source: PLoS One. 2015 Oct 15;10(10):e0140370. doi: 10.1371/journal.pone.0140370 (PMC4607301; doi:10.1371/journal.pone.0140370)

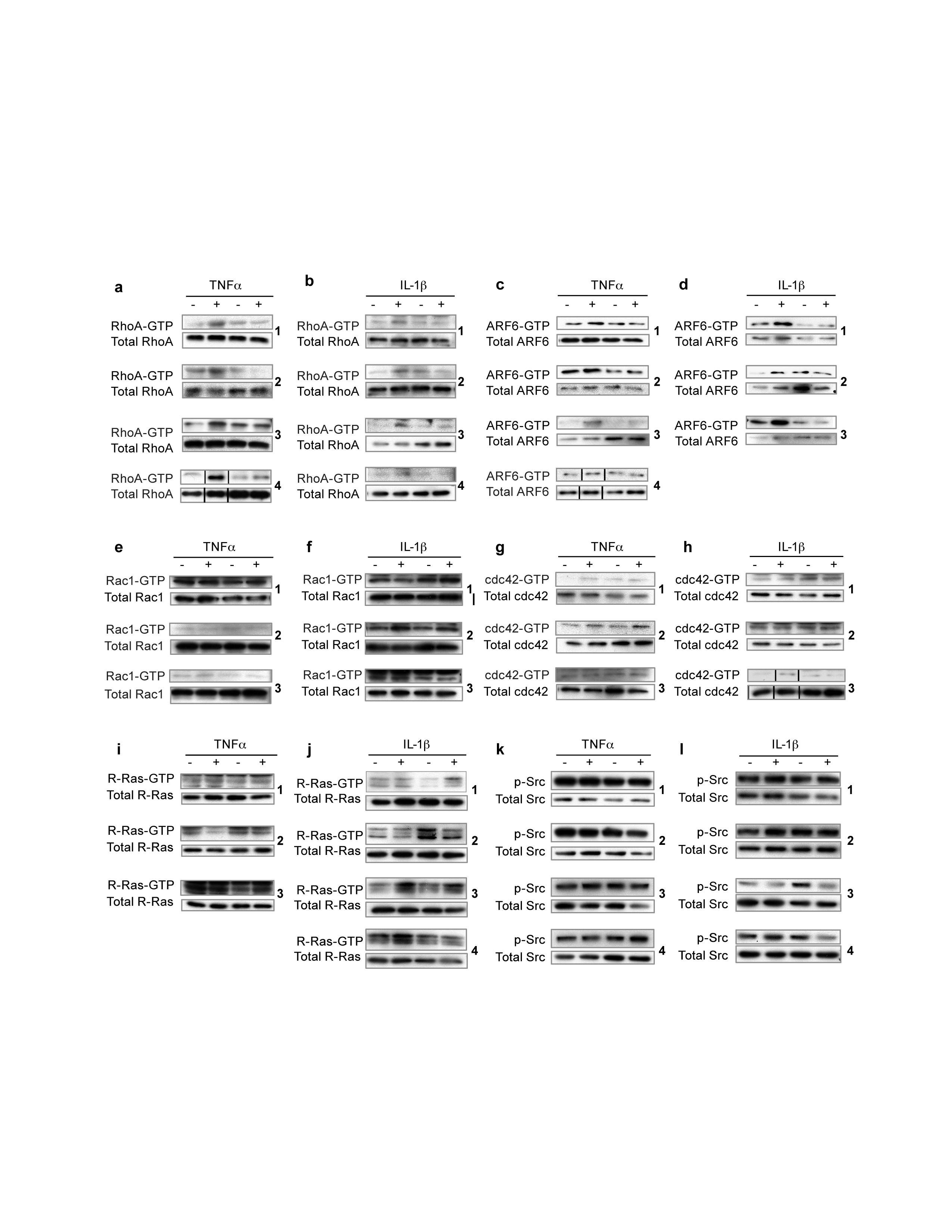

Supplement: S1 Fig — Endothelial cells were exposed to 10 μM D3 or 7-DHC in combination with 2ng/mL TNF-α or IL-1β. Lysates were analyzed for RHOA-GTP and ARF6-GTP levels using appropriate precipitation assays. (TIF) [file pone.0140370.s001.tif]

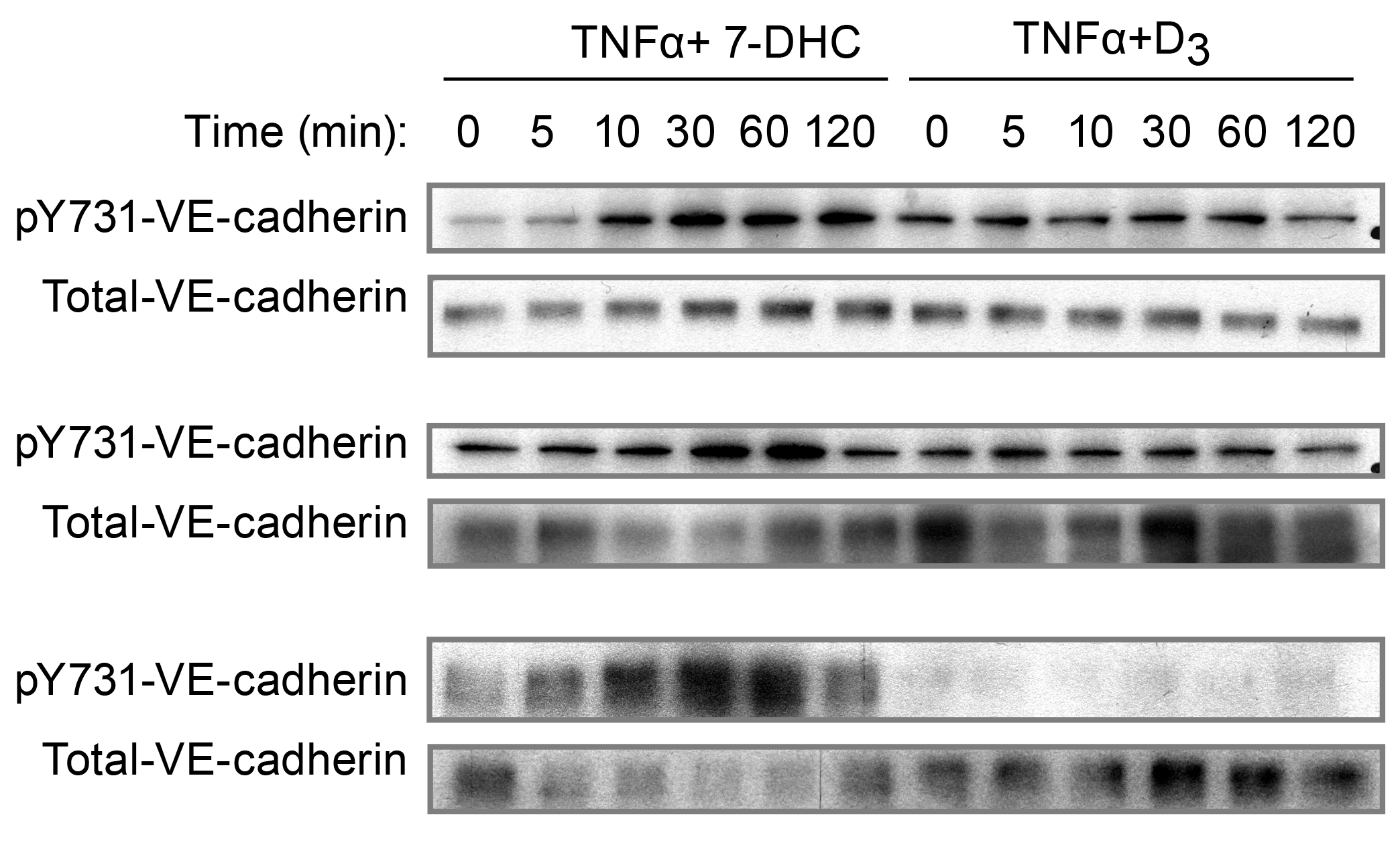

Supplement: S2 Fig — Endothelial cells were treated with TNF-α and either 7-DHC or D3 for the denoted times and lysates were immunoblotted for p731 VE-cadherin or total VE-Cadherin. (TIF) [file pone.0140370.s002.tif]
